# Neuro-autonomic changes induced by remote ischemic preconditioning (RIPC) in healthy young adults: Implications for stress

**DOI:** 10.1016/j.ynstr.2019.100189

**Published:** 2019-07-27

**Authors:** Igor Khaliulin, Arnold N. Fleishman, Nadezhda I. Shumeiko, TatyanaV. Korablina, Stanislav A. Petrovskiy, Raimondo Ascione, M.-Saadeh Suleiman

**Affiliations:** a Bristol Medical School, University of Bristol, Level 7, Bristol Royal Infirmary, Upper Maudlin Street, Bristol, BS2 8HW, UK; b Research Institute for Complex Problems of Hygiene and Occupational Diseases, 23 Ulitsa Kutuzova, Novokuznetsk, Kemerovo Oblast, 654041, Russia; c Information Technology Department, Siberian State Industrial University, Ulitsa Kirova, 42, Novokuznetsk, Kemerovo Oblast, 654007, Russia

**Keywords:** Remote ischemic preconditioning, Heart rhythm variability, Autonomic nervous system, ANS, autonomic nervous system, BP, blood pressure, DBP, diastolic blood pressure, DFA, detrended fluctuation analysis, HF, high frequency, HR, heart rate, HRV, heart rate variability, LF, low frequency, RIPC, remote ischaemic preconditioning, SBP, systolic blood pressure, VLF, very low frequency

## Abstract

The mechanisms underlying the protective effects of remote ischemic preconditioning (RIPC) are not presently clear. Recent studies in experimental models suggest the involvement of the autonomic nervous system (ANS) in cardioprotection. The aim of this study was to investigate the changes in ANS in healthy young volunteers divided into RIPC (n = 22) or SHAM (n = 18) groups. RIPC was induced by 1 cycle of 4 min inflation/5 min deflation followed by 2 cycles of 5 min inflation/5 min deflation of a cuff placed on the upper left limb. The study included analysis of heart rate (HR), blood pressure (BP), heart rate variability (HRV), measurements of microcirculation and porphyrin fluorescence in the limb before and after the RIPC. RIPC caused reactive hyperemia in the limb and reduced blood porphyrin level. A mental load (serial sevens test) and mild motor stress (hyperventilation) were performed on all subjects before and after RIPC or corresponding rest in the SHAM group. Reduction of HR occurred during the experiments in both RIPC and SHAM groups reflecting RIPC-independent adaptation of the subjects to the experimental procedure. However, in contrast to the SHAM group, RIPC altered several of the spectral indices of HRV during the serial sevens test and hyperventilation. This was expressed predominantly as an increase in power of the very low-frequency band of the spectrum, increased values of detrended fluctuation analysis and weakening of correlation between the HRV parameters and HR. In conclusion, RIPC induces changes in the activity of ANS that are linked to stress resistance.

## Highlights

• Brief ischemia/reperfusion episodes of distant organs (RIPC) protect other organs.
• Mechanism of RIPC is not known but it involves neuronal activity.
• RIPC applied to volunteers was interspersed with mild mental and physical stress.
• RIPC was confirmed by hyperemia in the limb and metabolic response to hypoxia.
• Heart rate variability shows that RIPC modulates ANS to increase stress resistance.

## 1 Introduction

The mammalian body can recruit powerful innate mechanisms that are highly effective at protecting tissues and organs against detrimental events including ischemia and reperfusion (I/R) injury. One example is a phenomenon known as remote ischemic preconditioning (RIPC). Experiments on animals showed that the heart can be protected against I/R injury by applying several short cycles of ischemia and reperfusion to a tissue or organ that is remote from the heart (Gaspar et al., 2018). In addition to the heart, RIPC may also confer protection of other organs such as the brain (Tülü et al., 2015), lung (Li et al., 2014; Weber et al., 2018), kidney (Gassanov et al., 2014; Zarbock et al., 2015), intestine (Li et al., 2013) or skeletal muscle (Addison et al., 2003; Kocman et al., 2015).

Due to its simplicity and non-invasiveness nature, RIPC has attracted a lot of interest especially from clinical colleagues. Several clinical trials have confirmed that applying RIPC to one of the limbs (arm or leg) can be cardioprotective in different clinical settings (Cheung et al., 2006; Hausenloy et al., 2007; Heusch, 2018; Hoole et al., 2009; Thielmann et al., 2013; Venugopal et al., 2009; Zhou et al., 2010). However, some of the recent clinical trials have not shown any added cardioprotective benefits (Hausenloy et al., 2016; McCrindle et al., 2014; McDonald et al., 2014). A likely explanation for this controversy could be heterogeneity of the patient population and different nature of the clinical intervention(s) (Barsukevich et al., 2014; Pickard et al., 2015). More importantly, the mechanisms responsible for the RIPC-induced protection have not been fully addressed (Pickard et al., 2015). Understanding these mechanisms is critical in the search for mediators that will enable us to achieve optimal protection. Although reports have indicated the involvement of humoral, neural and systemic mechanisms in RIPC (Heusch et al., 2015; Pickard et al., 2016; Ravingerova et al., 2016), the actual pathway (signal) connecting the remote tissue to the target has yet to be identified.

One important mechanism implicated in mediating RIPC involves the autonomic nervous system (ANS). Experiments on animals revealed an increase of parasympathetic vagal activity resulting in the RIPC-induced reduction of infarct size (Aulakh et al., 2017; Gourine and Gourine, 2014; Hausenloy and Yellon, 2007). It has also been shown that the autonomic reflex pathway activation, which may occur in RIPC, is the result of triggering an endogenous protective mechanism (Mastitskaya et al., 2012). Similar conclusions were made based on the finding that vagus nerve stimulation mimics the cardioprotective effect of RIPC (Donato et al., 2013). The neurogenic pathway of the RIPC application is complemented by the production of endogenous vasodilators, such as adenosine and bradykinin, associated with activation of calcitonin gene-related peptide (Schoemaker and van Heijningen, 2000; Steensrud et al., 2010; Wolfrum et al., 2005; Kleinbongard et al., 2017) and opioid peptides (Ravingerova et al., 2016). Interestingly, recent work of Lieder et al. has demonstrated a tight link between vagus and an unidentified humoral factor released by the spleen in the protective mechanism of RIPC (Lieder et al., 2018). It is assumed that acetylcholine released by vagus nerve affects the heart through muscarinic receptors (Steensrud et al., 2010). We have also recently shown that ANS is involved in cardioprotection by RIPC in a mouse model (Abdul-Ghani et al., 2017). However, little work has been done in humans either in health or with disease (Loukogeorgakis et al., 2005; Tsai et al., 2004). Investigating this in healthy subjects has the advantage of avoiding excessive experimental heterogeneity. In this context, heart rate variability (HRV) analysis represents a very useful tool as an effective non-invasive way to assess regulation of cardiac function, which has been used to study ANS activity (Electrophysiology, 1996). The aim of the present investigation was to study the possible involvement of the neural mechanisms in RIPC. For this purpose, we carried out HRV analysis in resting condition and under mild mental (serial sevens test) and motor stress (hyperventilation), as well as limb microcirculation and metabolism using laser Doppler imaging in healthy young individuals.

## 2 Methods

### 2.1 Ethical approval

All experiments were carried out in Novokuznetsk, Russia, at the Research Institute for Complex Problems of Hygiene and Occupational Diseases. A total of 40 young volunteers were recruited in this study (age range 18–23years) and informed about the predefined objectives. All volunteers signed the “Declaration of informed consent”. Studies conformed to the standards set by the Declaration of Helsinki adopted by the 64th WMA General Assembly (Fortaleza, Brazil, October 2013), and the procedures have been approved by the Ethical Committee of the Research Institute for Complex Problems of Hygiene and Occupational Diseases, (Novokuznetsk, Russia).

**Fig. 1:**
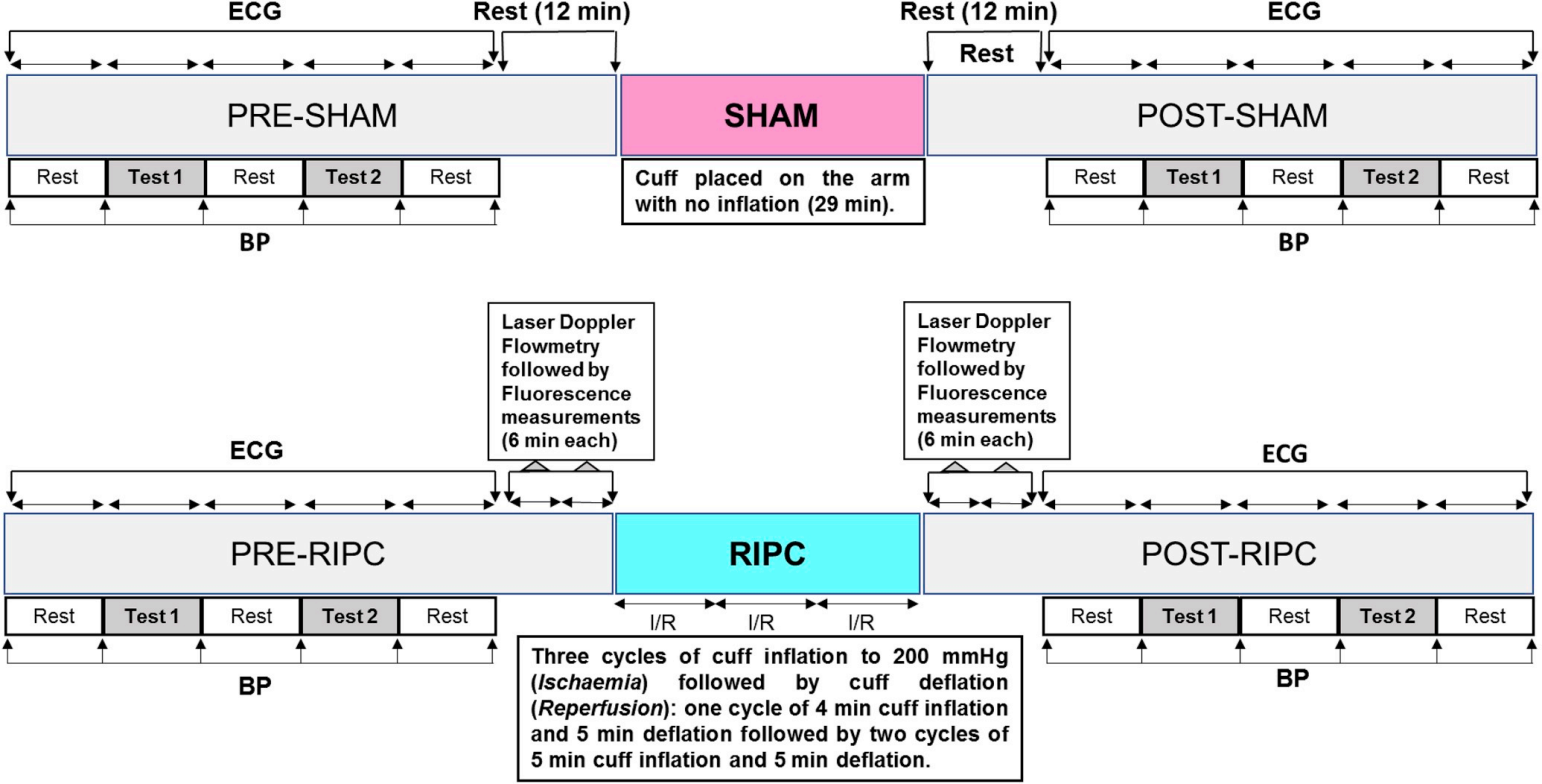
**Outline of the experimental protocol.** Test 1: serial sevens test; Test 2: hyperventilation; BP: blood pressure. Duration of ECG recording during the serial sevens test, hyperventilation and the three periods of rest was 256 RR intervals (approximately 3.6 min each). BP was measured at the end of each period of ECG recording. The Doppler diagnostics was not performed in the SHAM group.

The volunteers were recruited for either RIPC (RIPC group, n = 22) or control sham (SHAM group, n = 18). The two groups were matched for gender with nine females in each group. The following selection/exclusion criteria were applied for all volunteers of both RIPC and SHAM groups. The volunteers were examined by a neurologist and a general practitioner and those who had somatic or neurological complaints were excluded. Those with acute respiratory disease, smoking, on medication or consumed alcohol the day before were also excluded from the study. None of the volunteers had any form of diabetes. All measurements were carried out during the period 11.00 a.m.–13.00 p.m. The volunteers were fed at least 2 h prior to the experiment. The lead 1 of a three lead ECG was used for monitoring heart rate (HR) and for HRV analysis since it gives good reading of the R wave. Blood pressure cuff connected to sphygmomanometer was used to apply RIPC and to measure systolic (SBP) and diastolic (DBP) blood pressure. Microcirculatory limb blood flow and metabolism were assessed using laser Doppler flowmetry and fluorometry. The data acquisition was performed according to the protocol shown in Fig. 1 and described in detail below.

### 2.2 Experimental protocol

Volunteers from the RIPC group were exposed to two mild stress tests (serial sevens and hyperventilation) before and after RIPC interventions (Fig. 1). The same tests, which were interspersed with rest periods, were also performed at similar time points in the SHAM group. The duration of each stress test was equivalent to 256 RR intervals on the ECG (approximately 200 s; see explanation in 2.3.). The same duration was also allocated to the rest periods in between the tests. The ECG was recorded during each of the test and the rest periods (5 times before and 5 times after RIPC or SHAM interventions). At the end of each period (test or rest), blood pressure was measured using a sphygmomanometer with a cuff fastened on the upper left limb.

Prior to starting the RIPC protocol, volunteers had laser Doppler blood flow and fluorescence measurements, each of which lasted for 6 min (12 min in total). These measurements were also repeated immediately following RIPC application.

#### 2.2.1 Details of the mild stress tests

The serial sevens test is designed to cause mental load involved subtracting serial sevens starting from 500 (Abhishekh et al., 2014; Ruesch, 1944). The hyperventilation test involves deep diaphragm breathing over a period of 200 s. These tests allow assessing reaction of the subject to mild stressors and revealing adaptive reserves of the autonomic regulation of cardiac function (Chen et al., 2001; Fleishman, 1999; Kox et al., 2011; Van De Borne et al., 2001).

#### 2.2.2 Application of remote ischemic preconditioning (RIPC)

Each of the three cycles of RIPC involved inflation and deflation of the cuff fastened on the upper left limb. The pressure in the cuff was elevated to 200 mmHg. The duration of the first cuff inflation (ischemia) was 4 min, followed by 5 min of deflation (reperfusion). During the second and the third cycles, 5 min cuff inflation was followed by the 5 min of deflation. The total duration of the RIPC procedure was 29 min.

Laser Doppler imaging (see 2.4 for more details) was used in the RIPC group for analysis of microcirculation and measurements of the porphyrin level in the left limb (Fedorovich, 2012).

### 2.3 ECG & analysis of heart rate variability (HRV)

The HRV and HR analysis was performed using ECG recordings (lead 1 of the 3-lead ECG). An ECG duration covering 256 RR intervals was used for each stage of the experimental protocol (test and rest periods) before and after RIPC application (Fig. 1). For the HRV analysis, Fast Fourier transform that converts a signal from its original domain (time) to a representation in the frequency domain was used. For its calculation, the number of measurements needs to be equal to 2 in the power of n (e.g. 64, 128, 256, 512, etc.) (Marple, 1987). In our work, with restricted time for ECG recording, 256 RR intervals appeared to be optimal. Furthermore, it has been shown that this number of measurements provides a smooth spectral estimate with clearly outlined peaks in low- and high-frequency bands (Singh et al., 2004). The HRV analysis included both traditional indicators (dispersion, Fast Fourier transform with windows) and a nonlinear indicator detrended fluctuation analysis (DFA) (Fleishman, 2009). DFA of heart rate variability is used to quantify the fractal scaling properties of short- (α_1_) and intermediate-term (α_2_) RR interval time series (Tulppo et al., 2001). Decrease in DFA reflects a change towards a more periodic heart rate under stress (Kovács et al., 2015). Since we used relatively short periods of ECG recording (256 RR intervals) for the HRV analysis, we calculated DFA-α_1_.

**Fig. 2:**
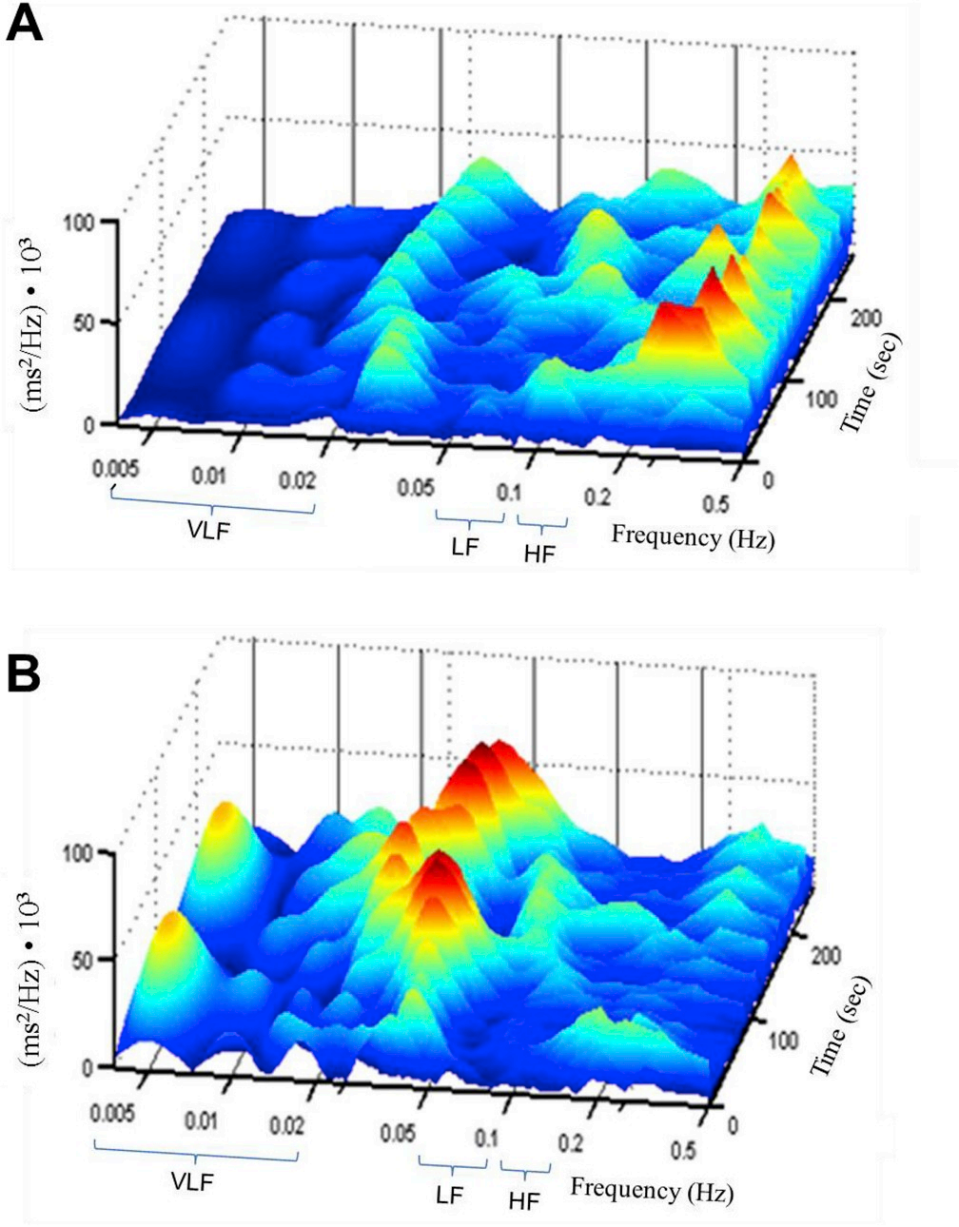
**Representative image of wavelet transform before and after RIPC.** ECG was recorded in a 24 years old healthy male volunteer. The wavelet transform was computed in this subject using RR intervals of ECG recorded before (A) and after RIPC (B) and presented as a distribution of power of oscillations (axis y) over time (axis z) at different frequencies (axis x). In this volunteer, RIPC resulted in the following changes of the calculated parameters of HRV: VLF increased from 410 to 633 ms^2^/Hz; LF increased from 72 to 235 ms^2^/Hz; HF was similar before and after RIPC (188 and 195 ms^2^/Hz, respectively).

Continuous wavelet transform was used for visualization and analysis of the spectral characteristics of HRV (Fig. 2). The Win Spectr v1.4-w/oReo Assembly 21032007 software (produced by Kurichev A.S., Novokuznetsk, Russia) was used for the HRV analysis. The wavelet transforms were based on the library CWTLib (http://cwtlib.sf.net/).

The following ranges of the analyzed HRV spectrum were selected for the frequency quantification (Fleishman et al., 2014): very low frequency (VLF), including VLF100 (0.005–0.015 Hz) & VLF50 (0.015–0.025 Hz), low frequency (LF, 0.08–0.12 Hz) and high frequency (HF, 0.15–0.35 Hz). The HF reflects the efferent vagal activity (Electrophysiology, 1996), *i.e.* activity of the lower (segmental preganglionic and ganglionic) neurons of parasympathetic system, whilst LF predominantly corresponds to the efferent activity of segmental and ganglionic neurons of sympathetic system. In contrast, data at VLF (100 & 50) reflect mainly the activity of suprasegmental centers of the ANS (sympathetic & parasympathetic centers located above the spinal cord) (Akselrod et al., 1985; Fleishman, 1999; Thayer et al., 2012). Additionally, the ratio (HF/LF) was used to assess changes in vagus activity relative to the activity of sympathetic system.

### 2.4 Laser Doppler flowmetry and fluorescence spectroscopy

**Fig. 3:**
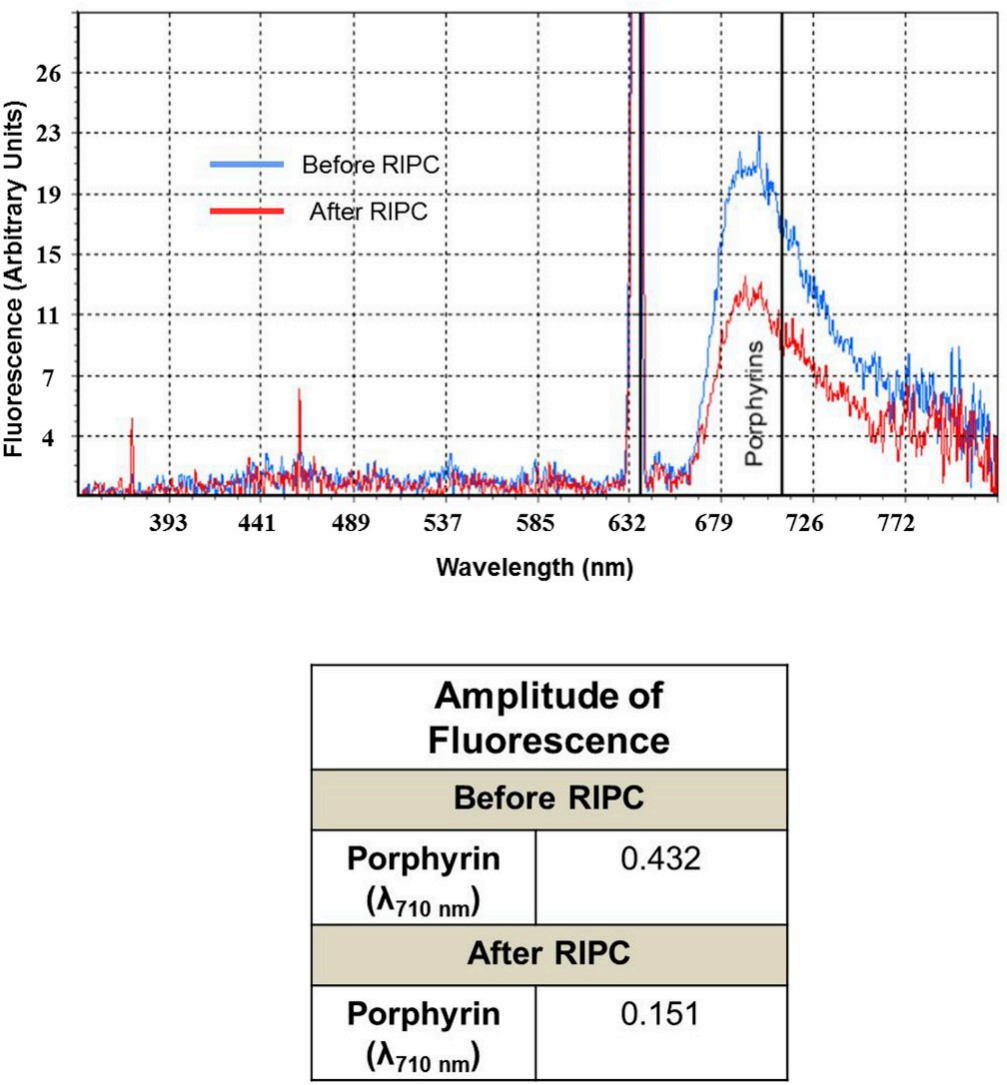
Representative changes in porphyrin level before and after RIPC. Porphyrin was measured in the left index finger using a laser Doppler flowmetry imaging system before and after application of RIPC. Fluorescence at the wavelength of 710 nm corresponded to the level of porphyrin in the blood. The values of porphyrin fluorescence measured in an 18 years old healthy female volunteer before and after RIPC are shown in the table.

The aim of these measurements was to evaluate the limb peripheral circulation and tissue metabolism using non-invasive laser technique (Hausenloy and Yellon, 2007). The changes in microcirculation in the upper limb and tissue metabolism were assessed prior to and after RIPC using a laser Doppler flowmetry imaging system (LACC-M, NPO “Lazma”, Russia) on the palmar surface of the left forefinger as described previously (Lakowicz, 2006) with the excitation wavelength of 530, 630 and 800 nm. The following parameters of microcirculation were assessed (Tikhomirova et al., 2012): MI – microcirculation index. MI = N_er_ • V_m_, where N_er_ is number of erythrocytes and V_m_ is mean velocity of erythrocyte flux. MP – the level of perfusion. This parameter represents the mean value of the MI and characterizes the value of perfusion. σ – mean square deviation. This index describes the temporal variability of perfusion. It reflects the average blood flow modulation in all frequency ranges; K_v_ – coefficient of variation. K_v_ = σ/МP · 100%. The increase in the value of K_v_ reflects improvement in microcirculation. MvT – micro vascular tone. MvT = σ • MBP/A_max_ • MP, where MBP is mean arterial blood pressure and A_max_ is maximal amplitude of perfusion oscillations. Rv – intravascular resistance. SI – shunting index. SI = A_act_/AM, where A_act_ is the amplitude of the predominant active factor of microcirculation control (neurogenic or endothelial) and AM is the amplitude of microcirculatory myogenic oscillations. Porphyrin level was assessed by measuring porphyrin autofluorescence at excitation/emission of λ = 630/710 nm (an example is shown in Fig. 3). Metabolic stress (e.g. chronic hypoxia) is associated with increase in laser-induced porphyrin fluorescence (Litvinova et al., 2009) whilst a positive resistance to stress is characterized by reduced level of porphyrin in the aerobic cells (Schwartsburd, 2001). It should be noted, that there might be differences between the level of metabolites and proteins in the fingertip and in the central blood vessels (Nishiumi et al., 2019). Therefore, the interpretation of these measurements should be considered with care.

### 2.5 Statistical analysis

**Fig. 4:**
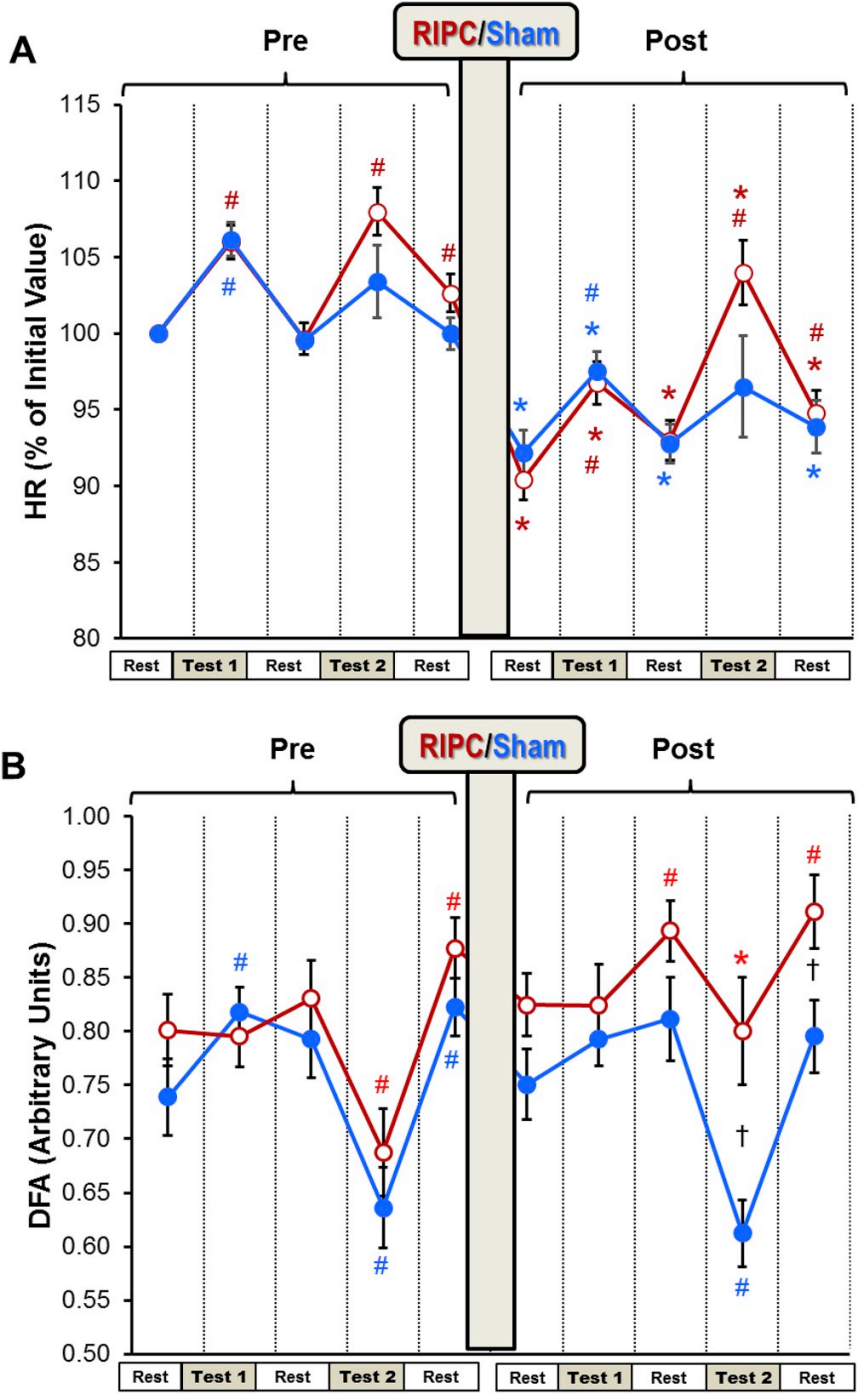
**The effect of RIPC on HR and DFA** Changes in HR (panel A) and DFA (Panel B) measured before and after RIPC (Red). HR was expressed as percentage of the rest value measured at the beginning of the experiment. The absolute values of HR can be found in Table 2. Different stages of the experimental protocol are outlined in the Methods and shown in Fig. 1. Data are presented as mean ± SEM (n = 22 and 18 for RIPC and SHAM). RIPC data are shown in red and SHAM are shown in blue. *P < 0.05 *vs*. corresponding indices before the RIPC/SHAM. #P < 0.05 *vs*. corresponding pre-test parameters. †P < 0.05 between the RIPC and SHAM groups. (For interpretation of the references to colour in this figure legend, the reader is referred to the Web version of this article.)

**Fig. 5:**
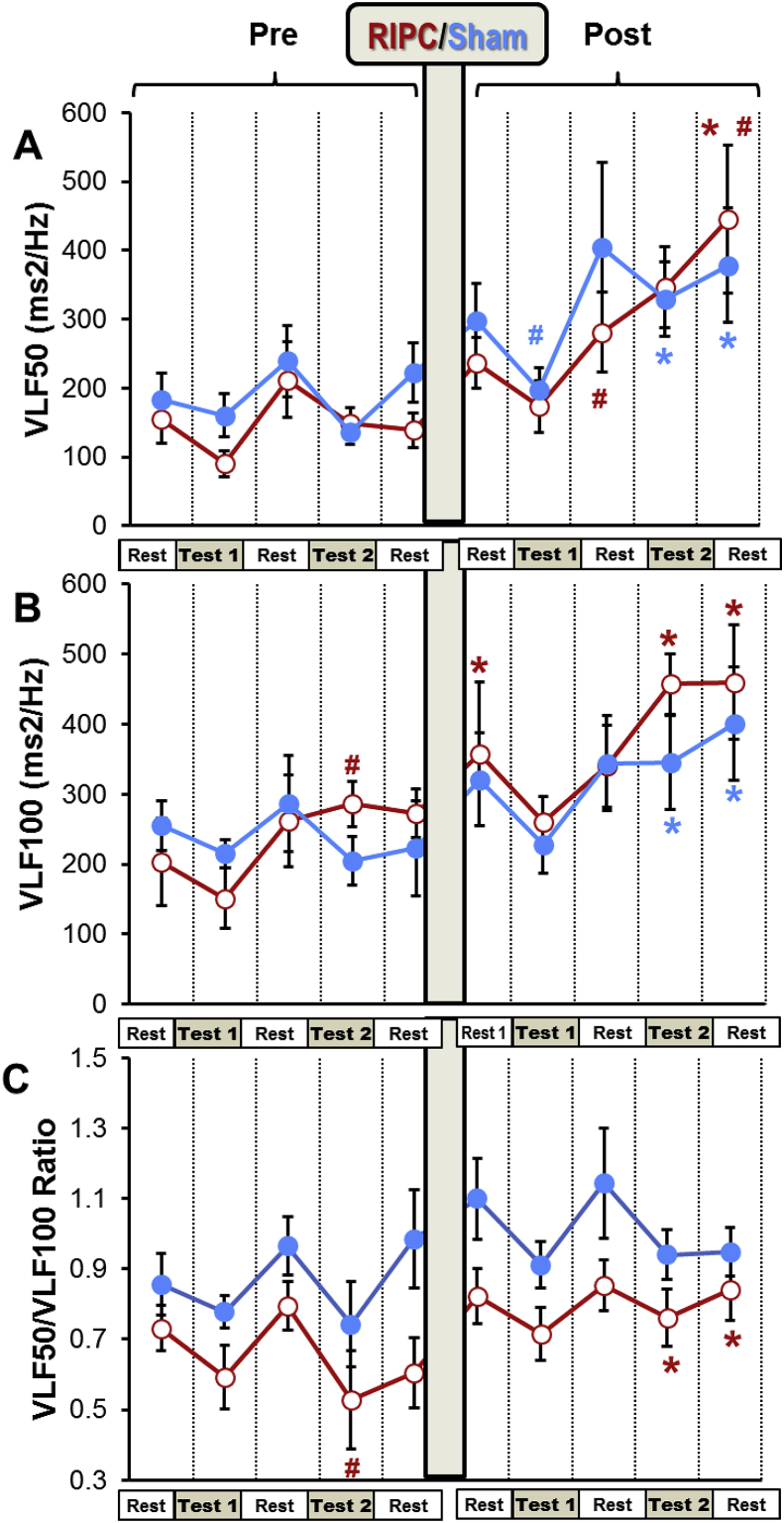
**The effect of RIPC on VLF.** Different stages of the experimental protocol are outlined in the Methods and shown in Fig. 1. Data are presented as mean ± SEM (n = 22 and 18 for RIPC and SHAM). RIPC data are shown in red and SHAM are shown in blue. *P < 0.05 *vs*. corresponding indices before RIPC/SHAM. #P < 0.05 *vs*. corresponding pre-test parameters. (For interpretation of the references to colour in this figure legend, the reader is referred to the Web version of this article.)

**Fig. 6:**
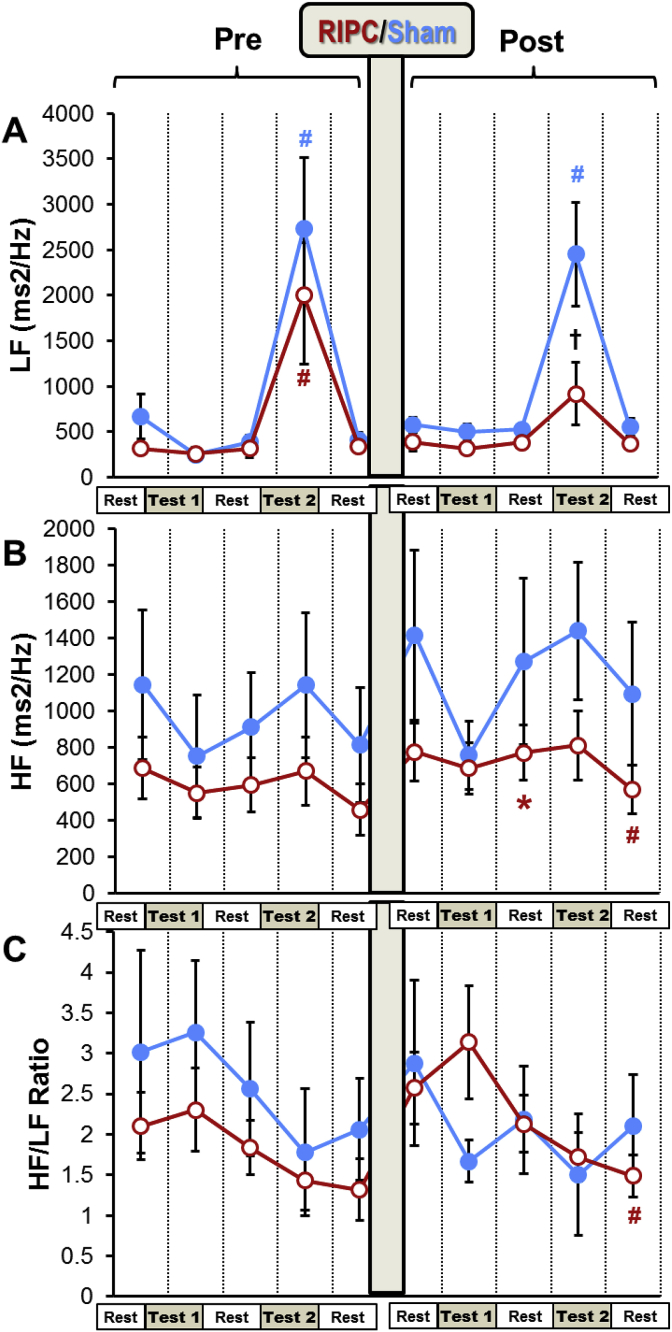
The effect of RIPC on LF and HF. Different stages of the experimental protocol are outlined in the Methods and shown in Fig. 1. Data are presented as mean ± SEM (n = 22 and 18 for RIPC and SHAM). RIPC data are shown in red and SHAM are shown in blue. *P < 0.05 *vs*. corresponding indices before the RIPC/SHAM. #P < 0.05 *vs*. corresponding pre-test parameters. †P < 0.05 between the RIPC and SHAM groups. (For interpretation of the references to colour in this figure legend, the reader is referred to the Web version of this article.)

All the data presented in the tables and in Fig. 4, Fig. 5, Fig. 6 are expressed as mean ± SEM. One-way ANOVA repeated measures followed by the LSD multiple comparison test was used to determine statistical significances of the differences between corresponding parameters measured at different time-points (during stress tests and the three resting periods before and after RIPC/SHAM) within each group. The statistical significances of the differences between the corresponding parameters of the RIPC and SHAM groups were analyzed using the two-tailed unpaired Student's t-test. Multiple Pearson correlations were computed between the spectral and non-linear HRV parameters (VLF (50 &100), LF, HF and DFA) and HR. The statistical analysis was performed using the software IBM SPSS Statistics, V. 24. Differences were considered significant where P < 0.05.

## 3 Results

### 3.1 Limb blood flow and tissue metabolic changes during RIPC

Table 1 shows the changes of blood flow and porphyrin level in the left limb in the RIPC group. As expected, three cycles of RIPC applications on the arm were associated with hyperemic changes in microcirculatory blood flow. In particular, there was a significant increase in mean square deviation (σ) and shunting index (SI). A significant reduction in porphyrin levels measured using the fluorescence spectroscopy was observed in this group (Table 1).

**Table 1:** Changes in the microcirculation indices before and after RIPC using Laser Doppler imaging.

Parameter	Pre-RIPC	Post-RIPC	% Change
σ	1.99 ± 0.24	3.38 ± 0.40 **	70
МP	22.46 ± 3.36	18.29 ± 1.71	−19
MvT	3.70 ± 0.41	4.79 ± 0.60	29
R_v_	0.26 ± 0.02	0.20 ± 0.02	−23
SI	1.68 ± 0.21	2.09 ± 0.17 *	24
Porphyrins 710 nm	0.31 ± 0.02	0.21 ± 0.01 ***	−32

MP – the level of perfusion, σ – mean square deviation, MvT – micro vascular tone, Rv – intravascular resistance, SI – shunting index. Values are Mean ± SE. *(Р<0.05); **(P < 0.01); ***(P < 0.001) vs. Pre-RIPC.

### 3.2 The effect of RIPC on BP and HR

The average BP values (SBP and DBP) did not change over the experiment in both SHAM and RIPC groups (Table 2). Prior to RIPC application, there was a significant increase of HR in both groups in response to the serial sevens test and to the hyperventilation in the RIPC group (Fig. 4A). However, after RIPC (or equivalent period in SHAM), HR decreased significantly in both groups prior to the tests. Both tests again resulted in increased HR compared to the corresponding pre-test (rest) values. However, they remained significantly lower than HR measured for these tests before RIPC (Fig. 4A, Table 2).

**Table 2:**
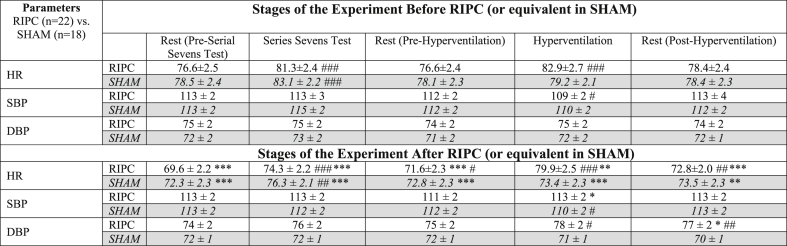
Changes in HR and BP before and after RIPC/*SHAM*.

Results of the experiments of RIPC group are presented in normal font on the white background; results of the experiments of SHAM group are presented in *Italic* font on the grey background.

* Р<0.05, **P < 0.01, ***P < 0.001 *vs.* corresponding indices Pre-RIPC.

#P < 0.05, ##P < 0.01, ###P < 0.001 vs. corresponding Background.

### 3.3 The effect of RIPC on the spectral indices of HRV

All the pre-SHAM HRV parameters were similar to the corresponding pre-RIPC indices of HRV (Fig. 4, Fig. 5, Fig. 6). Hyperventilation resulted in a significant fall in DFA in both groups (Fig. 4B). Very low frequency 100 (VLF100) was increased in the RIPC group by the hyperventilation test and returned to the pre-test level during the resting period (Fig. 5A). Hyperventilation also increased LF in the SHAM and RIPC groups by 4 and 6-fold, respectively (Fig. 6A).

DFA levels fell markedly during the two hyperventilation tests in the SHAM group. However, in the RIPC group, this index became significantly higher than in the SHAM group during the second hyperventilation test. Furthermore, in contrast to the SHAM group, after RIPC, DFA was increased during the rest following the serial sevens and hyperventilation tests compared to the pre-tests resting period (Fig. 4 B). An increase in VLF100 and VLF50 was observed during the post-RIPC/SHAM period in both groups, but this growth was higher in the RIPC group (Fig. 5). Thus, in the SHAM group, these parameters became significantly elevated only at the end of the experiment (during and after hyperventilation), whilst in the RIPC group, VLF100 was increased significantly over the whole post-RIPC period. VLF100 and VLF50 in the RIPC group grew by 2.3- and 2.9-fold at the end of the experiment, whilst in the SHAM group, this growth was less obvious (by 1.6- and 2-fold, respectively). Hyperventilation produced after RIPC/SHAM elevated LF in the SHAM group by 6-fold, but in the RIPC group, only by 1.6-fold. As a result, LF in this group became significantly lower than in the SHAM group at this time-point (Fig. 6A). HF was increased during the post-mental load (serial sevens test) resting period after RIPC compared to pre-RIPC, but not after the SHAM (Fig. 6B). However, during the last resting period, this parameter in the RIPC group became lower than during the pre-tests rest. RIPC tended to increase the HF/LF ratio during the serial sevens test but fell significantly by the end of experiment compared to the pre-tests rest. This was not seen in the SHAM group (Fig. 6C).

### 3.4 Correlation analysis of HR and the spectral indices of HRV

Correlation between the pre-SHAM parameters of HRV and HR were similar to those of pre-RIPC (data not shown).

The action of RIPC was reflected not only in the values of the spectral characteristics of HRV but also in the correlations between different indices calculated during the five periods of pre-RIPC and at the corresponding periods of post-RIPC (Table 3).

**Table 3:** Multiple Pearson correlation between the parameters of HR and HRV before and after RIPC.

Before RIPC*Rest (Pre-Serial Sevens Test)*										After RIPC *Rest (Pre-Serial Sevens Test)*								
		HR		VLF100	VLF50			LF	HF		HR	VLF100	VLF50			LF	HF	
HR		1								HR	1							
VLF100		−0.51*		1						VLF100	−0.45*	1						
VLF50		−0.41*		0.90**	1					VLF50	−0.46*	0.79**	1					
LF		−0.33		0.52**	0.39			1		LF	−0.24	0.54**	0.68**			1		
HF		−0.58**		0.84**	0.78**			0.54**	1	HF	−0.71**	0.69**	0.62**			0.62**	1	
DFA		0.31		−0.16	−0.02			−0.64**	−0.40*	DFA	0.38	−0.19	−0.20			−0.52*	−0.40*	
*Serial Sevens Test*										*Serial Sevens Test*								
		HR		VLF100	VLF50			LF	HF		HR	VLF100	VLF50			LF	HF	
HR		1								HR	1							
VLF100		−0.35		1						VLF100	−0.39	1						
VLF50		−0.49*		0.64**	1					VLF50	−0.31	0.86**	1					
LF		−0.30		0.76**	0.80**			1		LF	−0.16	0.33	0.38			1		
HF		−0.71**		0.61**	0.65**			0.54**	1	HF	−0.57**	0.16	0.22			0.40	1	
DFA		0.56**		−0.46*	−0.23			−0.32	−0.66**	DFA	0.30	0.14	0.15			−0.50*	−0.63*	
*Rest (Post-Serial Sevens Test)*										*Rest (Post-Serial Sevens Test)*								
		HR		VLF100	VLF50			LF	HF		HR	VLF100	VLF50			LF	HF	
HR		1								HR	1							
VLF100		−0.58**		1						VLF100	−0.54**	1						
VLF50		−0.52*		0.87**	1					VLF50	0.93**	0.79**	1					
LF		−0.29		0.60**	0.78**			1		LF	−0.11	0.54**	0.62**			1		
HF		−0.76**		0.71**	0.54**			0.43*	1	HF	−0.61**	0.63**	0.60**			0.69**	1	
DFA		0.18		0.04	−0.06			−0.36	−0.42*	DFA	0.25	−0.05	0.05			−0.35	−0.49*	
*Hyperventilation*											*Hyperventilation*							
	HR		VLF100			VLF50	LF		HF		HR	VLF100		VLF50	LF			HF
HR	1									HR	1							
VLF100	−0.51*		1							VLF100	−0.47*	1						
VLF50	−0.34		0.77**			1				VLF50	−0.46*	0.78**		1				
LF	−0.57*		0.10			0.25	1			LF	−0.34	−0.10		0.13	1			
HF	−0.62**		0.75**			0.47*	0.62**		1	HF	−0.57**	0.51*		0.34	0.40			1
DFA	0.73**		−0.41*			−0.20	0.62**		−0.58**	DFA	0.49*	−0.05		0.26	−0.44*			−0.50*
*Rest (Post-Hyperventilation)*											*Rest (Post-Hyperventilation)*							
	HR		VLF100			VLF50	LF		HF		HR	VLF100		VLF50	LF			HF
HR	1									HR	1							
VLF100	−0.59**		1							VLF100	−0.44*	1						
VLF50	−0.69**		0.61**			1				VLF50	−0.36	0.93**		1				
LF	−0.37		0.05			0.53*	1			LF	−0.16	0.35		0.30	1			
HF	−0.74**		0.58**			0.64**	0.56**		1	HF	−0.62**	0.42*		0.37	0.65**			1
DFA	0.35		−0.01*			−0.25	−0.73**		−0.52**	DFA	0.21	0.27		0.38	−0.50*			−0.52**

VLF100 – very low frequency (0.005–0.015 Hz), VLF50 – very low frequency (0.015–0.025 Hz), LF – low frequency (0.08–0.12 Hz), HF – high frequency (0.15–0.35 HZ), DFA – detrended fluctuation analysis.

The correlation is calculated for RIPC group (n = 22). Statistical significance of the correlation: *P < 0.05, **P < 0.01.

VLF100 maintained a significant correlation with VLF50 throughout the experiment. The strong link between VLF100 and HF was observed during all five bouts of the ECG recording, including the two tests and three resting periods prior to RIPC (see experimental protocol depicted on Fig. 1), as well as a significant negative correlation between the two VLFs and HR. HR also significantly and positively correlated with DFA during the serial sevens test and hyperventilation. A strong positive correlation of HF with both VLF50 and LF appeared at all five periods of the ECG recording prior to RIPC.

After RIPC, correlation between the different HRV parameters and HR changed. Although the correlation during the pre-test measurements and the two resting periods after the tests looked similar to that of pre-RIPC, it significantly weakened during the tests (Table 3). Particularly noticeable was the weakening of correlation during the serial sevens test. Thus, during this test performed after RIPC, HR retained a significant correlation only with HF, and VLF100 lost correlation with all the parameters except VLF50. Likewise, VLF50 significantly correlated only with VLF100. LF lost correlation with the two VLFs and HF and retained a significant correlation only with DFA. Similarly, during hyperventilation carried out after RIPC, correlation between HR and LF, VLF100 and DFA, VLF50 and HF, as well as HF and LF became insignificant.

## 4 Discussion

The results of the HRV analysis combined with the data of laser Doppler diagnostics provide for the first-time data showing that RIPC initiates adaptive reaction, including the activation of both sympathetic and parasympathetic systems, which is likely to increase body resistance to stressors. These changes occur at both central suprasegmental centers and the lower, segmental preganglionic and ganglionic, levels of ANS and are manifested in reactive hyperemia, reduced metabolic stress (i.e. decrease in porphyrin levels), and more importantly, in changes in the HRV indices following RIPC in young healthy subjects. A reduction of HR in the course of the experiment reflect both adaptation of the subjects to the experimental procedure and parasympathicotonia caused by RIPC.

### 4.1 Changes of heart rate and blood pressure during the mild stress tests before and after RIPC

Stress tests (both serial sevens and hyperventilation) prior to RIPC were associated with significant increase in HR which was reversed during the resting periods. After RIPC, HR dropped by approximately 10%. This could be explained by activation of parasympathetic system induced by the three brief episodes of the limb ischemia/reperfusion (i.e. RIPC) (Donato et al., 2016). However, similar changes of HR were observed in the SHAM group after the rest corresponding to RIPC (Fig. 3A, Table 2). This finding implies that the experimental conditions themselves contributed to this reduction in HR. Obviously, our experimental settings, regardless of the presence or absence of the RIPC procedure, induced an adaptive reaction in the young healthy subjects manifested in the increased parasympathetic activity. Nevertheless, this finding does not rule out participation of parasympathetic system in the protective effects of RIPC, as seen from the analysis of HRV (see below).

There were little changes in BP throughout the experiment (Table 2) despite the changes in HR. The lack of change in BP can be explained by the fact that we used mild stress tests. During these tests, the BP homeostasis would be maintained and supported by the well-known central, local and humoral regulatory mechanisms (Hubner et al., 2015).

### 4.2 Changes in HRV indices and laser Doppler diagnostics indicate stress limiting effects of RIPC

The baseline HRV characteristics were similar in the RIPC and the SHAM groups indicating that the state of HR regulation in these groups was comparable at the beginning of the experiment. Consistent with previous reports (Fleishman, 2009), stress tests (serial sevens or hyperventilation) were associated with changes in HRV indices (Fig. 5, Fig. 6). Thus, increased LF observed prior to both RIPC and SHAM during hyperventilation could be related to the stress-induced activation of sympathetic system (Electrophysiology, 1996).

RIPC, in general, resulted in a considerable increase in power of the VLF bands of the HRV spectra. In particular, VLF100 increased significantly immediately after RIPC and continued growing throughout the post-RIPC period. The physiological significance of this range of frequencies still has to be investigated. However, it is now certain that VLF fluctuations of the HRV may serve as an indicator of the activity of suprasegmental (above the spinal cord) centers of the ANS (Akselrod et al., 1985; Fleishman, 1999; Thayer et al., 2012; Thayer and Lane, 2009). The VLF is associated with both sympathetic and parasympathetic systems as demonstrated by the findings that both the systemic parasympathetic muscarinic receptor blocker atropine (Taylor et al., 1998) and the acute ganglionic blockade of sympathetic system with hexamethonium (Radaelli et al., 2005) decreased VLF variability. We studied VLF at two bands of the HRV spectra, 0.005–0.015 Hz (VLF100) and 0.015–0.025 Hz (VLF50) which could reflect different mechanisms (Fleishman et al., 2014). Specifically, the results of our experiments show that after RIPC, the VLF50/VLF100 was elevated in the RIPC group during and after hyperventilation (Fig. 5C) *vs*. the corresponding pre-RIPC values, and this was accompanied by a decrease in LF (Fig. 6A). Since the reduction in LF corresponds to the diminished activity of sympathetic system (Electrophysiology, 1996), this may indicate that VLF50 reflects mostly activity of parasympathetic system whilst changes in VLF100 correspond to the changes in activity of sympathetic system. The increase in VLF band of the HRV spectra after the RIPC application suggests stress-limiting response because an increase in these oscillations has found to be associated with the resistance stage of the General Adaptation Syndrome (Fleishman, 2009).

After RIPC, hyperventilation significantly increased LF oscillations in subjects of the SHAM but not RIPC group indicating the reduced sympathetic response to this mild motor stress in the volunteers subjected to RIPC. The elevated vago-sympathetic index (HF/LF) during the mental test in this group after RIPC also points to the relatively increased parasympathetic tone. These changes were in keeping with the dynamics of DFA. Prior to RIPC, DFA was decreased during hyperventilation in both groups (Fig. 3B). This reflects a change towards a more periodic heart rate characteristic of the stress-related activation of sympathetic tone. (Kovács et al., 2015). However, after RIPC, the reduction in DFA in response to hyperventilation was significantly weakened in the RIPC group (Fig. 3B) indicating the reduced stress-reaction to this test after RIPC with a prevailing parasympathetic activity. These findings can be supported by the earlier reports showing that the effect of RIPC can be abolished by ganglionic blockade via systemic administration of hexamethonium (Gho et al., 1996) or by bilateral vagotomy (Basalay et al., 2012). Furthermore, vagus nerve stimulation was found to reduce myocardial injury (Calvillo et al., 2011; Katare et al., 2009; Mioni et al., 2005). Using the selective pharmacologic inhibition of vagal neurons, Mastitskaya et al. showed that RIPC cardioprotection depends on the activity of vagal pre-ganglionic neurons and consequently an intact parasympathetic drive (Mastitskaya et al., 2012). These neurophysiological studies clearly support a role for parasympathetic activity in the RIPC-induced protective effects.

Along with the discussed above changes of the HRV parameters pointing to activation of parasympathetic system, other changes indicate increased sympathetic activity after RIPC. Thus, the significant increase in HR during hyperventilation, reduced vago-sympathetic ratio during the rest following hyperventilation and increased level of VLF100 observed after RIPC are all indicative of the enhanced activity of the sympathetic system. The simultaneous activation of sympathetic and parasympathetic systems may seem paradoxical at first sight. However, it has been convincingly shown that the sympathetic and parasympathetic nervous systems are not “opposites”; rather, the interactions are complex (Goldberg et al., 1975; Heusch et al., 2015) and this was confirmed by others working on hypertensive rats (Berg and Jensen, 2011). It has been revealed that reduction in energy supply may lead to the decrease or even loss of the RIPC phenomenon, as it is observed in the elderly (Melillo et al., 2011; Moro et al., 2011). Based on these findings, it can be suggested that increased sympathetic activity after RIPC, seen in our study, is necessary to provide energy for the implementation of parasympathetic response.

The general characteristic of changes in the inter-frequency relationships induced by RIPC in the 22 subjects was a reduction in the degree of correlation between most of the parameters of cardiac rhythm and its variability during the stress tests and particularly during the serial sevens test when only three pairs of the parameters, VLF100-VLF50, HR-HF and HF-DFA, retained their correlation seen prior to RIPC (Table 3). Similar data were shown by others (Papasimakis and Pallikari, 2010) who demonstrated reduced correlation between the HRV parameters during relaxation in young healthy subjects. The observed changes induced by RIPC can be regarded as the conditions contributing to the strengthening of adaptive mechanisms in the body (Fleishman, 2009).

RIPC in our experiments brought about a significant reduction in the porphyrin levels measured in the limb subjected to the short-term episodes of I/R (Table 1). These results are in accord with a recent work of Oba et al. (2015) who found that in mice, RIPC produced by ischemia/reperfusion of a hind limb, reduced infarct size and increased serum erythropoietin, which would reduce porphyrin level (Thunell, 2000). It also has been shown that chronic hypoxia triggers a large and abnormal laser-induced porphyrin auto fluorescent signal from tissues (Litvinova et al., 2009) whilst stress tolerance is associated with attenuation of the level of porphyrin due to coordinated negative feedback reactions involving inhibition of hemeoxygenase 1 and 5-aminolevulinic acid synthase (Schwartsburd, 2001; Papasimakis and Pallikari, 2010; Papaioannou et al., 2013; Rolf, 1998). Consequently, reduction in porphyrin autofluorescence in our study is another confirmation of increased stress resistance induced by RIPC in young healthy subjects and this metabolic change is likely to be mediated by ANS (Oba et al., 2015).

In general, the changes in the HRV parameters, as well as the decrease in porphyrin autofluorescence, imply that RIPC triggers the adaptive mechanisms involving the ANS. The general stress-limiting effect of RIPC may explain its ability to protect a variety of organs and tissues against ischemia/reperfusion injury, as discussed in the Introduction. The increased body resistance to stressors in our experiments was characterized by parasympathicotonia with concomitant activation of the ergotropic sympathetic mechanisms. Inflammatory response could also be involved in this mechanism along with reaction of ANS, as shown by Papaioannou et al. (2013). It is also worth mentioning that the RIPC-induced protection can be transferred not only by the nervous system, but also via the humoral pathways, which may include nitric oxide, stromal derived factor-1α, microRNA-144, but also other, not yet identified factors (Heusch et al., 2015; Lieder et al., 2018).

### 4.3 Study limitations

The interpretation of the changes in HRV and laser Doppler characteristics during the mild tests and after the RIPC presented in our work are based on the well-established previously published data. However, some controversy has been mentioned in previous studies. For example, Breuer et al. (1993) have found that light physical exercises, when heart rate is increased to 100 beats/min, are not characterized by increased plasma catecholamine concentrations and both LF and HF are decreased. The authors concluded that this kind of exercises are associated with the reduced parasympathetic tone rather than activation of sympathetic system. Hopf et al. (1995) have questioned the opinion that LF band of the HRV spectra reflects the activity of sympathetic system because the preganglionic cardiac sympathetic blockade did not alter spectral power in the LF oscillations. However, we have to note that their study was conducted with segmental thoracic epidural anesthesia, which could compensatory increase the central sympathetic drive (Rolf, 1998), increase blood catecholamine level, and consequently affect the LF power of oscillations. The physiological meaning of the VLF band remains least investigated. Therefore, our conclusions still need to be correlated with and validated by more direct evaluation of the activity of the ANS, such as peroneal microneurography or plasma catecholamines measurements. This work has to be undertaken in the near future.

Another limitation of our work was the individual differences manifested in the variety of the structural HRV responses to RIPC and stress tests in different subjects. The individual differences may depend on numerous factors including the initial body state, fitness, sex *etc*. This resulted in rather high variability of the HRV parameters in our study where the number of subjects in each group was relatively small.

We assessed blood porphyrin autofluorescence in a fingertip using laser Doppler fluorometry and not in the central blood vessels. There are suggestions, including a recent study (Nishiumi et al., 2019), that the level of many but not all proteins and metabolites in the blood of the fingertip correlate well with their level in venous blood. Therefore, our data using this technique should be treated with caution.

## 5 Conclusions

In this work, RIPC represents an adaptation to a few episodes of limb ischemia and reperfusion resulting in the general systemic protective response to the stressors. This is expressed in the reduction of heart rate along with the increase in power of the VLF bands of the HRV spectra, increased DFA, weakening of correlations between the spectral parameters of HRV and reduction of porphyrin autofluorescence. The changes of VLF power of oscillations of HR in our study suggest an important role of central mechanisms, whilst HF and LF mostly reflect segmental mechanisms of autonomic relegation during RIPC. Activation of the parasympathetic centers of the CNS plays a major role in adaptive effect of RIPC, and sympathetic system supports their enhanced activity. This study may serve as a basis for developing effective non-invasive and pharmacological strategies of protection from a variety of stress conditions. Further studies of the algorithms and mechanisms of this effect of RIPC in elderly population and in different clinical settings is necessary in order to advance these strategies.

## Competing interests

The authors declare that they have no competing interests.

## Author contributions

I.K. contributed to conception of the study, analysis and interpretation of data, writing of the manuscript and its critical revision. A.N.F. was responsible for the conception and design of experiments, data acquisition and interpretation, drafting of the manuscript and its critical revision, as well as for preliminary medical examination of all the volunteers who took part in the study. N.I.S. contributed to acquisition and analysis of the data and critical revision of the manuscript; T.V.K. took part in data analysis and interpretation and in critical revision of the manuscript; S.A.P. contributed to acquisition, analysis and interpretation of the data and drafting of the manuscript; R.A. contributed to data interpretation and critical revision of the paper; M.S.S. contributed to conception, design, data interpretation, writing of the manuscript and its critical revision. All authors approved the final version of the manuscript. All authors agree to be accountable for all aspects of the work. The experiments were performed at the Research Institute for Complex Problems of Hygiene and Occupational Diseases, Novokuznetsk, Kemerovo Oblast, Russia.
